# Public Health in the Twenty-First Century: The Role of Advanced Technologies

**DOI:** 10.3389/fpubh.2014.00224

**Published:** 2014-11-10

**Authors:** Muni Rubens, Venkataraghavan Ramamoorthy, Anshul Saxena, Nancy Shehadeh

**Affiliations:** ^1^Robert Stempel College of Public Health and Social Work, Florida International University, Miami, FL, USA; ^2^College of Business, Florida Atlantic University, Boca Raton, FL, USA

**Keywords:** public health, megatrend, twenty-first century, nanotechnology, artificial intelligence

## Introduction

Nearly a century ago, Charles-Edward Amory Winslow, defined public health as “the science and art of preventing disease, prolonging life, and promoting health and efficiency through organized community effort” (1). From health policy reforms to scientific advances with many technological innovations, a range of forces are converging to cause a seismic shift in how public health is practiced. We think that in the twenty-first century, global public health system will be completely restructured. Reliance on complex technologies like nanotechnology and artificial intelligence (AI), which were previously only used in fields like computer science and physics, will become the new trend. Using such advanced technologies would become megatrends of public health in new and exciting ways. Historically, public health, in comparison to other scientific disciplines, has been behind the curve in terms of using advanced technologies (2). This article highlights the current trends in public health in terms of use of advanced technologies like nanotechnology and AI and how they can impact the future of public health in the twenty-first century.

## Nanotechnology and Public Health

### What is nanotechnology?

Nanotechnology is the science, which involves the design, synthesis and application of materials and devices, which have smallest functional organization on the nanometer scale in at least one dimension (3). According to Pautler and Brenner, “the most widely accepted definition of scale for nanotechnology is 1–100 nm” (4). For the purposes of advancing public health, there are several advantages to engineering materials on such a small scale. For example, using “nanomedicine” approaches allows researchers to apply drug therapies to human cancer cells with previously unachievable accuracy and fewer treatment related adverse effects (4). Other examples include biomedical technology that can monitor patient’s metabolic systems from inside the body, and nano-engineered bone prostheses that could be implanted with the highest achievable precision (5). Thus, the use of such complex medical technologies with individual based approaches could significantly improve population health over time with indirect advantages to public health.

## Nanotechnology in Public Health

### Protecting the human right to clean water

One specific use of nanotechnology, which can improve public health, is environmental nanotechnology, which can produce clean and safe drinking water for human consumption and use. In the modern world, availability of water continues to be a problem on an international level due to factors like global warming, drought, and unprecedented population growth (6). For example, nanotechnology can be used to make water-testing sensors to purify water for safe consumption (5–7). This can improve public health on a global level by reducing the numbers of people harmed or killed by health problems associated with unsanitary water (7). Furthermore, safe drinking water can help primary education in several developing countries in Africa, Asia, and Latin America, where children are often unable to attend schools due to the lack of clean water sources (7).

### Reducing the global disease burden

Nanotechnology could also improve public health through advanced drug administration strategies. For example, there are many cancer-fighting drugs such as Abraxane^®^ (for breast cancer) and Doxil^®^ (for ovarian cancer), which are “nanoenabled” and have already been approved by the Food and Drug Administration (FDA) (4). Nanotechnology is also being used to manufacture single dose regimens of hepatitis B vaccine with better penetrance and immune activation profiles compared to the currently administrated four dose regimen. This can be considered a major advance in public health because many more vaccines can be derived with single dose options thereby increasing compliance and effectiveness (4).

#### Safety concerns

Nanotechnology is already being used for manufacturing thousands of consumer products marketed globally. For example, nanoparticles are used in popular sunscreen brands such as Burt’s Bees^®^ and Coppertone^®^ (4). However, there are still questions about the long-term effects of nanotechnology on general health and environment. There are no FDA regulations within the U.S. that govern the use of such materials in consumer products. Many researchers have expressed concerns because safety controls and monitoring systems are lacking for nanotechnological products both within the U.S., and globally (5, 6, 8, 9).

## Artificial Intelligence and Public Health

### What is artificial intelligence?

Artificial intelligence, often abbreviated as “AI,” is defined as “the science and engineering of making intelligent machines, especially intelligent computer programs. It is related to the task of using computers to understand human intelligence, but AI does not have to confine itself to methods that are biologically observable” (10). Broadly speaking, AI involves the creation of “intelligent” systems capable of performing complex data analyses, which are superior to the ones performed by existing data systems without the use of AI.

## Artificial Intelligence in Public Health

The use of AI for public health spans back to the 1960s, when professionals from disciplines like computer science, chemistry, and biology began using AI programs in complex medical researches (11). Several researchers convened at the conference *Artificial Intelligence in Medicine in Europe* (AIME) in 2007 to discuss the role of AI in modern medicine, and their opinions were collectively published in the year 2009 (11). In our opinion, AI can best serve the goals of public health only with cross-disciplinary expansions and collaborations. This entails not only the development of computer science but also incorporating advancements from fields like epidemiology, biology, genetics, modern medicine, and public health policy.

### Artificial intelligence in disease surveillance

According to Neill (12), public health surveillance may be defined as “the process of detecting, characterizing, tracking, and responding to disease outbreaks, other health threats (such as a bioterrorist attack, radiation leak, or contamination of the food or water supply), and other patterns relevant to the health of populations (such as obesity, drug abuse, mental health, or malnutrition).” This type of surveillance is conducted at multiple levels, ranging from local community levels all the way up to global community health settings (12, 13). It also spans across several types of public and private entities such as government agencies, healthcare clinics, hospitals, pharmacies, and health corporations (13).

National Institutes of Health in the U.S. has several ongoing studies targeting the use of AI for improving surveillance of both communicable and non-communicable diseases (13). Modern disease surveillance systems use AI to help automate the process of disease surveillance so that there is less reliance on human labor; to make the process more expedited so that real-time and predictive surveillance can occur; to allow for data to come from a much wider variety of sources than traditional systems; and to allow the results of such data to be disseminated to public health officials and the general public on a wider and faster basis (14). A new type of system has emerged specifically for using digital data to improve public health surveillance. This has been referred to as “digital surveillance” and it “attempts to provide knowledge of public health issues by analysis of health information stored digitally, and the distribution and patterns governing access to these data” (15). Data gathered from Internet sources used to feed a digital surveillance system is one such advancement. Milinovich et al. (15) offer many examples of such Internet-based health surveillance systems. These include tracking communicable and non-communicable diseases, mental health trends, illegal drug use trends, and the impact of health policies. Disease surveillance systems using AI can help in tracking health issues as they occur and predict health issues in the future. For example, one such system was able to predict a rise in U.S. flu cases approximately 1–3 weeks before they occurred and was based on Yahoo search queries for related health information variables (16). In addition to data from search engines, data from social media have also been used to feed digital surveillance systems. For example, Lee et al. (17) built a flu and cancer surveillance system, which relies on Twitter data to give real time estimates of trends such as severity of disease, progression, symptoms being experienced, and treatments being implemented.

There is significant evidence to state that Internet-based disease surveillance systems offer advantages over traditional disease surveillance systems. Traditional disease surveillance systems are typically fed by data from public health officials working in hospitals and agency settings, whereas Internet-based systems can offer more data over different, and oftentimes larger population segments (16). Systems such as “Google Flu Trends” have predicted flu outbreaks earlier than traditional disease surveillance systems. However, there are some disadvantages to these systems as well because it relies on data provided by people who have access to Internet and use it for health information. In spite of advanced web delivery options, Internet use continues to be low in many underdeveloped and developing nations. Also, there are privacy concerns over the use of data within these systems (16). While such systems may offer advancements to modern day disease surveillance, Polgreen et al. (16) argues that “they do not have the capacity to replace traditional surveillance systems and they should not be viewed as an alternative, but rather an extension” to traditional disease surveillance systems. Our opinion is that these systems should be integrated into traditional systems in the coming years.

When opining about the future of AI for disease surveillance, Neill states that this field is now experiencing a “major paradigm shift” because compared to the past, today’s surveillance systems are relying on ever increasing volumes of data and data from non-traditional sources like online news feeds and Twitter feeds (12, 15).

## Other Technologies

There are several other technologies that hold promise for advancing the public health in the twenty-first century. Some of the major ones include mHealth (mobilehealth), virtual reality, biosensors for healthcare, disappearing technology, etc. mHealth is defined as, “the practice of medicine and public health, supported by mobile communication devices for health services and information” (18). mHealth and e-health are widely used in interventions for adult and pediatric obesity (18, 19). Another promising technology includes virtual reality, where a computer generated advanced interface could simulate a real word scenario and communicate the healthcare advice. For example, virtual reality enhanced cognitive behavioral therapy was more effective in weight reduction strategies when compared to other strategies like inpatient multimodal treatment and standard cognitive behavior therapy (20). The use of biosensors could revolutionize public health delivery in the twenty-first century. Micro-miniaturized sensors could be permanently implanted in biological fluids for continuous evaluations of biomedical parameters in diabetes, cancers, and cardiovascular diseases (21). Through the use of yet another modern application known as disappearing technology, public health could expand to dimensions hither to unknown. This technology uses bio/eco-resorbable chips implanted into the body. Though majority of the applications of this technology are currently limited to clinical medicine, public health prospects could be subsequently researched (22).

Table 1 shows how advanced technologies can impact essential public health services.

**Table 1:** **Core areas of public health impacted by advanced technologies**.

Essential public health services	Examples of role of technologies
Monitor health status to identify community health problems	Uses artificial intelligence to collect, manage, integrate, and display health profile databases
Diagnose and investigate health problems and health hazards in the community	Use artificial intelligence for faster clinical decision-making of communicable diseases to prevent epidemics
Inform, educate, and empower people about health issues	Current electronic health record (EHR) systems captures only clinical data. An adapted EHR (aEHR) that include multiple factors, including individual characteristics, the community, the environment, and a host of social and psychological factors could help in developing public policy and informing and empowering people about health issues. aEHR systems could be used to expand patient education opportunities. Among the benefits of good patient education are “improved self-reported health status, lower health-care costs, increased health knowledge, shorter hospitalizations, and less frequent use of health-care services” (26). aEHR could be used to make customized health information and messages based on individual patient’s clinical data. These tailored messages could be send directly to specific patients. One such message could be reminders to take medicine or vaccines
Mobilize community partnerships to identify and solve health problems	Decision support systems of EHR, including reminders and alerts, could be expanded (aEHR) to include not only clinicians but also community groups and policy makers. One such example is the epidemic monitoring system. Such surveillance system could gather data from various sources and predict trends and alert local, state and even national health departments if the incidents of any disease surpasses a pre-programed threshold
Develop policies and plans that support individual and community health efforts	Policies to use nanotechnology for safe drinking water and single dose vaccines with increased compliance
Enforce laws and regulations that protect health and ensure safety	Nanotechnology-based gas sensors to monitor pollution (27). These sensor units are portable and provide accurate and instantaneous environmental pollution levels. They can be easily installed to broadcast real-time data on pollution data servers and offer topological outline of the monitored locations. Law enforcement agencies can use this information to detect the sources of pollution and implement containment measures. Such integrated systems would also be beneficial in generating public awareness and directing policy initiatives for polluted areas
Link people to needed personal health services and assure the provision of health care when otherwise unavailable	Mobile-health (mHealth) is the use of mobile computing and communication technologies for improving health. Systematic review and meta-analysis of studies on mobile technology used interventions have shown that mHealth are effective in linking people to health services (24)
Assure a competent public health and personal healthcare workforce	A study by Salinas-Miranda et al. (25) have shown that using technology-enhanced authoring tools, learning management systems, survey research software, online communities of practice, and mobile communication are highly effective in training the public health workforce
Evaluate effectiveness, accessibility, and quality of personal and population-based health services	Dulin et al. (23) has shown that geographic information systems (GIS), analytical hierarchy process (structured techniques to make complex decisions), and multi-attribute assessment (analyzes multiple attributes and combines data elements to positively influence decision making) can be used to easily and effectively evaluate access to primary care services
Research for new insights and innovative solutions to health problems	Innovative solutions and cutting-edge research on technologies like tele-health and health information technology will advance public health in twenty-first century

## Conclusion

We think that there should be increased awareness among public health professionals and policy makers about the use of advanced technologies to improve health care and address newer health challenges. This would also lead to improved and widespread implementation of modern technologies. The continued use and advancement of such technologies cannot be isolated within the dimensions of abstract science. It should be directed toward public awareness for increased support and funding. The researchers and practitioners of these technologies rely on the public support, and therefore, these technologies should be oriented toward the public whom they intend to serve. Indeed, Currall et al. (28) have shown that public perceptions about tools, such as nanotechnology, play a vital role in how these technologies progress. They argue that “understanding public sentiment toward any new innovation is pivotal because, historically, public perceptions, and attitudes have shaped the direction and pace of scientific activity in a number of fields.” Today’s populace is very supportive for scientific and technological progress. Thus, we predict that public health will continue to advance into the twenty-first century and take full advantage of today’s popular support. It is clear that dedicated research efforts, driven by public education will establish a megatrends of highest use of technology for improved public health outcomes.

## Conflict of Interest Statement

The authors declare that the research was conducted in the absence of any commercial or financial relationships that could be construed as a potential conflict of interest.
